# Phylogeny matters: revisiting ‘a comparison of bats and rodents as reservoirs of zoonotic viruses’

**DOI:** 10.1098/rsos.181182

**Published:** 2019-02-13

**Authors:** Cylita Guy, Jeneni Thiagavel, Nicole Mideo, John M. Ratcliffe

**Affiliations:** 1 Department of Ecology and Evolutionary Biology, University of Toronto, 25 Willcocks Street, Toronto, ON, Canada M5S 3B2; 2 Department of Biology, University of Toronto at Mississauga, 3359 Mississauga Road, Mississauga, ON, Canada L5L 1C6

**Keywords:** trait-based approaches, zoonotic disease, viral richness, reservoir species, bats, rodents

## Abstract

Diseases emerging from wildlife have been the source of many major human outbreaks. Predicting key sources of these outbreaks requires an understanding of the factors that explain pathogen diversity in reservoir species. Comparative methods are powerful tools for understanding variation in pathogen diversity and rely on correcting for phylogenetic relatedness among reservoir species. We reanalysed a previously published dataset, examining the relative effects of species' traits on patterns of viral diversity in bats and rodents. We expanded on prior work by using more highly resolved phylogenies for bats and rodents and incorporating a phylogenetically controlled principal components analysis. For rodents, sympatry and torpor use were important predictors of viral richness and, as previously reported, phylogeny had minimal impact in models. For bats, in contrast to prior work, we find that phylogeny does have an effect in models. Patterns of viral diversity in bats were related to geographical distribution (i.e. latitude and range size) and life history (i.e. lifespan, body size and birthing frequency). However, the effects of these predictors were marginal relative to citation count, emphasizing that the ability to accurately assess reservoir status largely depends on sampling effort and highlighting the need for additional data in future comparative studies.

## 1. Introduction

In recent years, viruses carried by wild mammals have caused a number of severe human outbreaks (e.g. severe acute respiratory syndrome (SARS) [1], Ebola [2], Rabies [3]). These zoonotic, or species jumping, pathogens account for the majority of emerging infectious diseases worldwide [4], taxing global economies and causing significant human hardship. Among groups of mammalian species capable of carrying zoonotic viruses, bats (order Chiroptera) receive substantial attention. They act as reservoirs for pathogens such as, but not limited to, SARS, Middle Eastern respiratory syndrome coronavirus (MERS), Nipah, Hendra, Marburg, Ebola and Rabies [5–12]. The propensity of this mammalian group to host chronic and persistent viral infections, with few overt signs of disease, may make bats unique in their role as viral reservoirs [13–15]. Several studies have sought to identify ecological traits that explain patterns of viral diversity in bats [16–20] and have compared bats to other reservoir groups [21,22]. Although this body of work has sometimes yielded conflicting results about the importance of different ecological traits in driving patterns of viral diversity, it strongly indicates that bats are indeed ‘special’: they host more viruses per species than most other major mammalian orders, including rodents [21,22].

One of the first attempts to assess the idea that bats are unique as reservoirs for zoonotic viruses was a now widely cited study published by Luis *et al.* in 2013 (hereafter referred to as Luis *et al.*) [21]. The authors compiled data on viruses hosted by bats, which they then compared to data for rodents, another major zoonotic reservoir group. Luis *et al.* examined which aspects of host ecology explained the propensity of bat and rodent species to carry zoonotic viruses using a trait-based phylogenetic comparative approach. This study provides key support for the idea that bats are special as zoonotic reservoirs, because they host more zoonotic viruses per species than rodents. It also identifies key bat ecological traits that correlate with increased zoonotic viral diversity, e.g. bat species with smaller litters, larger body masses, greater longevity, more litters per year and geographical distributions overlapping with many other bat species (sympatry) carry a greater number of zoonotic viruses. It has since provided the basis for additional studies (e.g. [19,20,23]) and has informed and strengthened our understanding of bats as unique viral reservoirs.

In interspecies comparisons, phylogenetic methods have become commonplace to control for the statistical non-independence of species due to shared common ancestry [24,25]. Closely related species tend to share similar traits and some outcome of interest (e.g. pathogen diversity) could be improperly ascribed to those shared traits if phylogenetic relationships among species are not accounted for during analyses. Although the presence of phylogenetic signal (i.e. the degree to which trait variation is predicted by relatedness [26–28]) in species' traits and across clades is not ubiquitous [29,30], previous work has shown strong links between phylogeny and pathogen diversity in other mammalian groups [31]. Intriguingly, however, Luis *et al.* found that phylogeny explained little of the residual variation in their models predicting viral diversity in bats and rodents. The authors note that this was surprising, considering the strength of phylogenetic signal in other species’ traits [21]. Although the presence of phylogenetic signal in individual traits does not necessitate phylogenetic non-independence of the residuals in a regression model [32], the lack of effect of phylogeny in [21] is unexpected given its importance in other bat comparative studies (e.g. [33–36]). Additionally, phylogeny has been shown to predict patterns of viral sharing among bat species [23,37]. Therefore, considering the strong ties between host species' relatedness and viral transitions, phylogeny may still be expected to explain some of the residual variation in models predicting viral diversity.

Given the importance of phylogeny in other comparative studies involving bats, and the lack of phylogenetic signal found by Luis *et al.*, we reanalysed the data used in their study, applying more recent comparative methodologies and phylogenies*.* Luis *et al*. [21] used phylogenetic generalized least-squares regression (PGLS) [38], implemented with the Bininda-Emonds *et al.* [39] phylogeny (hereafter referred to as BE), to examine trait correlates of viral diversity in bats and rodents. We also implemented PGLS, but first considered how controlling for phylogeny during preliminary data transformations (i.e. principal component analysis (PCA)) impacts the variation explained in correlated life-history traits. Using our phylogenetic principal components, we then re-ran all analyses presented in Luis *et al.* using the same BE [39] phylogeny. However, given that the BE [39] phylogeny contains several polytomies (i.e. species relationships that are not fully resolved into dichotomies) and that topological inaccuracies can impact comparative methods [40], we also re-ran all PGLS models using more recent phylogenies for both bats [41] and rodents [42] that resolve these polytomies. We hypothesized that the use of phylogenetic PCAs and incorporation of updated phylogenies would reveal an effect of phylogeny in models examining ecological trait correlates of viral diversity in bats and rodents. Further, we hypothesized that changes in the amount of residual variation explained by phylogeny could shift the relative importance of ecological traits that predict viral richness in these groups.

## 2. Material and methods

### 2.1. Species’ traits and phylogenetic signal

All data used in our analyses were taken from electronic supplementary material, table S14 of Luis *et al*. [21]. As in that paper, we analysed the same species' traits: body mass, lifespan, number of litters per year, litter size, torpor use (categorized as no evidence of torpor use, some torpor use or true hibernation [21]), migration (for bats only; categorized as sedentary, regional migrants or long-distance migrants [21]), geographical distribution area, latitude of geographical range midpoint, number of species in the same taxonomic order that are sympatric (i.e. species with overlapping geographical ranges) and number of citations as of 2013 on Web of Science. All of our analyses were phylogenetically informed and carried out using comparative packages in R [43], to control for the statistical non-independence between related species [24,25]. To correct for phylogenetic non-independence in the residuals of our models, we used phylogenetic generalized least square regressions by restricted maximum likelihood [38] with a lambda (*λ*) model (see §2.2 below) and, depending on the analysis, one of three phylogenetic trees [39,41,42]. That is, in addition to the BE [39] phylogeny used in [21], we also used two more up-to-date, recently published phylogenies in our analyses [41,42]. To facilitate comparison with [21], we also used Pagel's lambda (*λ*) to calculate phylogenetic signal in the individual species' traits listed above (where *λ* was restricted to 0 ≤ *λ* ≤ 1).

### 2.2. Luis reanalysis with phylogenetically controlled PCAs

Like Luis *et al.* [21], we performed a PCA on correlated life-history traits (logged body mass, maximum longevity, number of litters per year and litter size). The novelty of our approach, however, was that our PCA was phylogenetically informed to control for relatedness among species [44]. Failing to control for phylogeny during a PCA can bias statistical estimators and lead to spurious results, even when phylogenetic comparative methods are applied in subsequent analyses [44]. For each analysis described below, we used the phylogenetic tree (i.e. BE [39]; Shi & Rabosky [41], hereafter S&R; or Faurby & Svenning [42], hereafter F&S) for bats, rodents or the combined bat and rodent data and computed phylogenetic principal component (pPC) scores in the original species space using *phytools* v. 0.5–20 [45]. For all pPCAs, pPC1 explained more than 99% of the life-history trait variation across species (Results and electronic supplementary material, tables S4 and S5), while pPC2 and pPC3 collectively explained less than 0.001% of trait variation. Thus, we included only pPC1 in subsequent analyses.

To examine which species’ traits correlate with either number of zoonotic viruses (i.e. those viruses capable of infecting humans) or total number of viruses in the bat and rodent species included in Luis *et al.* [21], we ran phylogenetic generalized least square regressions (PGLS) by restricted maximum likelihood [38] with Pagel's lambda using the R package *caper* v. 1.0.1 [46]. We re-ran all models for bats, rodents and the bat/rodent combined data presented in Luis *et al.* (i.e. table 1, electronic supplementary material, tables S2, S4, S8, S9 from [21]) using the same BE phylogeny used in their study [39]. The only difference was that our PC1 was phylogenetically informed, resulting in pPC1.

**Table 1.** Comparison of best PGLS models with number of zoonotic viruses as the response. Best PGLS models from our analyses are displayed alongside best results from the original Luis *et al.* paper. The phylogeny used to calculate estimates of *λ* in analyses is also indicated.

	this study				Luis *et al*. [21]			
phylogeny used	model	*R*^2^	*λ*	*p*-value	model	*R*^2^	*λ*	*p*-value
rodents								
Bininda-Emonds	∼cit. + rat sympatry	0.35	0.000	<0.0001	∼cit. + rat sympatry + IUCN	0.36	0.000	<0.0001
Faurby & Svenning	∼cit. + torpor	0.30	0.000	<0.0001				
	∼cit. + area	0.30	0.000	<0.0001				
	∼cit. + rat sympatry	0.30	0.000	<0.0001				
	∼cit. + IUCN	0.29	0.000	<0.0001				
bats								
Bininda-Emonds	∼cit. + latitude + area	0.14	0.806	0.0029	∼cit. + bat sympatry + PC1_b_	0.49	0.000	<0.0001
Shi & Rabosky	∼cit. + pPC1_b_	0.16	1.000	<0.0001				
Combined								
Bininda-Emonds	∼cit. + taxon sympatry	0.32	0.607	<0.0001	∼order × taxon sympatry + cit. + torpor	0.44	0.000	<0.0001
Faurby & Svenning	∼order + cit. + taxon sympatry + torpor	0.29	0.262	<0.0001				

We ranked all PGLS models using corrected Akaike information criterion (AIC_c_) scores and assumed that models with AIC_c_ scores that differed by less than or equal to 2 units had similar support [47]. Where multiple models had similar support, we adopted the principal of parsimony and assumed that the best model was the one with the fewest parameters [47]. Additionally, for each model, we calculated the adjusted coefficients of determination (*R*^2^).

### 2.3. Luis *et al*. reanalysis with pPCA and updated phylogenies

Using PGLS, we re-ran all models presented in Luis *et al.* [21] (following the methods outlined in §2.2 above) with more recent phylogenies for bats and rodents. Topological inaccuracies are known to bias other comparative methods [40], and the previously used BE [39] phylogeny is a composite phylogeny which contains several polytomies for both bats and rodents. Since 2013, improved phylogenies for both mammalian orders have been published that not only resolve most of these polytomies but are also based on more robust sequence data. For bats, we used the most comprehensive phylogeny currently available [41]. For rodents and the bat/rodent combined data, we used a recent species-level phylogeny for all extant and late Quaternary extinct mammals [42]. We tested for normality in variables that were not transformed in [21] and log-transformed those found to have skewed distributions. Specifically, for bats, in addition to mass and number of citations (as in [21]), we also log-transformed litter size, litters per year and range size, while for rodents, and when bats and rodents were considered together, we log-transformed these same variables and sympatry. As outlined in §2.2 above, we also ran a phylogenetically controlled PCA (i.e. pPCA) on correlated life-history traits for bats, rodents and the combined bat/rodent data and included pPC1 in the analyses.

### 2.4. An alternative modelling framework

Multi-collinearity between explanatory variables is common in ecological datasets. Many statistical methods are sensitive to collinearity and failing to address the relationships between variables can bias statistical inference [48]. To deal with collinear life-history traits, Luis *et al.* [21] employ a PCA. However, several bat and rodent ecological traits not included in this PCA are also correlated (electronic supplementary material, figures S4–S6). In addition to PCA, there are a variety of other approaches for dealing with collinear terms in multivariate analyses, such as the use of variance inflation factors (VIFs) [49]. VIFs do not require traits to be combined as in PCA, allowing for the effects of individual predictors in regression models to be assessed. In this final analysis, we employ the use of VIFs to remove correlated ecological traits. Whereas, in the above analyses, we look to replicate the methods of Luis *et al.* [21] while enacting changes to their implementation of phylogenetic comparative methods, in this set of analyses, we build on their work by adopting a different modelling approach.

**Figure 1.**
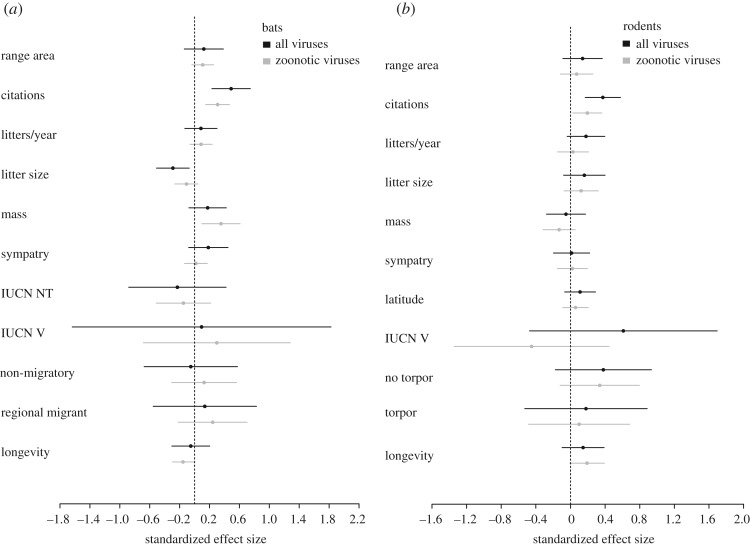
Plots of standardized effect size from PGLS models including all ecological traits with variance inflation factors less than or equal to 5 (instead of pPC1). Black dots represent the standardized effect size of traits for models examining correlates of total viral richness in bats (*a*) and rodents (*b*). Grey dots represent standardized effect size of traits for PGLS models of zoonotic viral diversity in bats (*a*) and rodents (*b*). Error bars represent 95% confidence intervals. The dashed line indicates an effect size of zero.

Prior to running PGLS models, we examined the distribution of our response variables (i.e. total number of viruses and number of zoonotic viruses) for bats, rodents and the bat/rodent combined data. We log-transformed these response variables to reduce skewness and checked subsequent PGLS models to ensure that residuals were homogeneous and normally distributed [50]. We then standardized all of our continuous ecological traits. Following [50], we used VIFs to check for collinear variables in our initial models for bats, rodents and the bat/rodent combined data. As in [51,52], we excluded any variable that had a VIF greater than 5. For bats, we removed latitude and torpor use from the initial model. For rodents, we retained all variables, and for the bat/rodent combined data, we removed order. Using the remaining ecological traits, we ran PGLS models for bats, rodents and the bat/rodent combined data, with either number of log-transformed zoonotic or log-transformed total viruses as the response. As in §2.3 above, we use the same variable transformations and the S&R [41] and F&S [42] phylogenies. For this set of models, we did not use a model selection procedure. Instead, we included all ecological predictors in the final model and assessed their relative effect sizes [53]. In addition to the *caper* package [46] used above to construct PGLS models, we also used the *phylolm* package v. 2.6 [54] to obtain additional information from PGLS models (i.e. 95% confidence intervals for relative effect size plots, figure 1 and electronic supplementary material, figure S7).

## 3. Results

### 3.1. Species’ traits and phylogenetic signal

For both bats and rodents, we found significant phylogenetic signal in many species' traits (rodents: 6 of 11 traits; bats: 6 of 12 traits; bat/rodent combined data: 7 of 11 traits) using the BE [39] phylogeny (electronic supplementary material, tables S1–S3). Using the S&R [41] and F&S [42] phylogenies, we also found significant phylogenetic signal in the majority of species traits (rodents: 6 of 11 traits, bats: 8 of 12 traits, bat/rodent combined data: 8 of 11 traits) (electronic supplementary material, table S1–S3). The presence of significant phylogenetic signal in most species traits is consistent with the results of Luis *et al.* (electronic supplementary material, table S13 from [21]).

### 3.2. Luis reanalysis with phylogenetically controlled PCAs

In all of our phylogenetic PCAs, the first phylogenetic principal component (pPC1) explained more than 99% of the life-history trait variation across species for bats, rodents and the bat/rodent combined data, regardless of the phylogeny used (electronic supplementary material, tables S4 and S5). This is in contrast with Luis *et al.* where the first three principal components (from a PCA that was not phylogenetically informed) accounted for 88% of the variance in bat life-history strategies and 93% of the variance in rodent life-history strategies [21]. The first four PCs accounted for 51.1% of the variance in life-history traits in the bat/rodent combined data [21]. Using the BE [39] tree, pPC1 for bats corresponded to increases in number of litters per year and litter size but decreases in body mass and lifespan (electronic supplementary material, table S4). For rodents, pPC1 represented increases in number of litters per year and decreases in body mass, longevity and litter size (electronic supplementary material, table S4). Finally, for the combined bat and rodent data, mass and longevity loaded negatively with pPC1, while number of litters per year and litter size loaded positively with pPC1, although the value for litter size was very close to 0 (electronic supplementary material, table S4).

Reanalysis of the Luis *et al.* data, using the same phylogeny as in the original paper [39], but including phylogenetically informed PCAs, slightly altered correlates of zoonotic and total viral diversity for bats, rodents and the bat/rodent combined data (tables 1 and 2). For bats, using [39], our best model for number of zoonotic viruses contained citations, latitude and range area (table 1 and electronic supplementary material, table S10). Both citations and range area had positive relationships with zoonotic viral richness, while latitude had a negative relationship (electronic supplementary material, table S11). Our best model for total number of viruses contained citations and sympatry (table 2 and electronic supplementary material, table S22), which both had positive relationships with viral richness (electronic supplementary material, table S23). These models differ slightly from the best models identified for bats by Luis *et al*. [21], which contained citations, sympatry and PC1. Further, phylogeny explained a large amount of additional residual variation in our bat PGLS models (electronic supplementary material, tables S10 and S22).

**Table 2.** Comparison of best PGLS models with total number of viruses as the response. Best PGLS models from our analyses are displayed alongside best results from the original Luis *et al.* paper. The phylogeny used to calculate estimates of *λ* in analyses is also indicated.

	this study				Luis *et al*. [21]			
phylogeny used	model	*R*^2^	*λ*	*p*-value	model	*R^2^*	*λ*	*p*-value
rodents								
Bininda-Emonds	∼cit. + rat sympatry	0.45	0.000	<0.0001	∼cit. + rat sympatry + torpor	0.46	<0.01	<0.0001
Faurby & Svenning	∼cit. + torpor	0.40	0.000	<0.0001				
	∼cit. + area	0.38	0.000	<0.0001				
	∼cit. + rat sympatry	0.38	0.000	<0.0001				
bats								
Bininda-Emonds	∼cit. + bat sympatry	0.28	0.522	<0.0001	∼cit. + bat sympatry + PC1_b_	0.48	0.000	<0.0001
Shi & Rabosky	∼cit. + latitude + area	0.32	1.000	<0.0001				
combined								
Bininda-Emonds	∼order + torpor + cit. + taxon sympatry	0.45	0.000	<0.0001	∼order × taxon sympatry + cit. + torpor	0.26	<0.01	<0.0001
Faurby & Svenning	∼order + cit. + taxon sympatry + torpor	0.40	0.000	<0.0001				

For rodents, using [39], our best model for total number of viruses and number of zoonotic viruses per species contained citations and sympatry (table 2 and electronic supplementary material, tables S6 and S18). This was in contrast to [21], where the best model for number of zoonotic viruses contained citations, sympatry and IUCN status (table 1) and the best model for total number of viruses contained citations, sympatry and torpor use (table 2). In both of our models, citation count and sympatry had positive relationships with viral diversity (electronic supplementary material, tables S7 and S19). Similar to [21], phylogeny did not explain any additional residual variation in our rodent PGLS models.

Reanalysis of the combined bat/rodent data using [39] altered correlates of viral diversity. In [21], best models for number of zoonotic and total viruses carried by species contained citations, torpor use and the interaction between order and taxon sympatry. Our best model for total number of viruses contained citations, sympatry, order and torpor use (table 2 and electronic supplementary material, table S26). Model coefficients indicate that rodent species carried fewer viruses than bats, citations and sympatry had positive effects on viral diversity, and that torpor use correlated negatively with viral diversity (i.e. true hibernators and species with some evidence for torpor use carried fewer viruses). Conversely, our best model for number of zoonotic viruses carried by species contained only citations and sympatry (table 1 and electronic supplementary material, table S14). Similar to models presented above, citations and sympatry had positive relationships with zoonotic viral diversity. In all of our bat/rodent combined models, phylogeny explained more additional residual variation than in [21] (electronic supplementary material, tables S14 and S26).

### 3.3. Luis reanalysis with pPCA and updated phylogenies

As outlined above in §3.2, pPC1 explained more than 99% of the life-history trait variation across species for bats, rodents and the bat/rodent combined data using the updated S&R [41] and F&S [42] phylogenies (electronic supplementary material, table S5). Using the S&R [41] phylogeny, for bats, pPC1 corresponded to decreases in longevity, mass and litter size, and increases in number of litters per year (electronic supplementary material, table S5). For rodents, using the F&S [42] phylogeny, pPC1 represented decreases in mass and longevity and increases in number of litters per year and litter size (electronic supplementary material, table S5). Finally, in the combined bat/rodent data, pPC1 (calculated using [42]) corresponded to increases in number of litters per year and decreases in mass, longevity and litter size (electronic supplementary material, table S5). Overall, using the new phylogenies resulted in minor changes to the loadings of pPCs relative to the loadings of pPCs calculated with the BE [39] phylogeny (see §3.2 above). However, these changes were more in line with partitioning species of bats and rodents into those with life histories that are fast (i.e. smaller body size, increased litter size and litters per year, decreased longevity) or slow (i.e. larger bodied, longer lifespans, fewer offspring and litters per year).

For bats, using the more recent S&R [41] phylogeny and pPCA altered correlates of both zoonotic and total viral diversity (tables 1 and 2). In their models for zoonotic and total viral diversity, Luis *et al.* found sympatry, citations and PC1 to be important [21]. Our best model for number of zoonotic viruses contained citations and pPC1 (table 1 and electronic supplementary material, table S12), which both had positive relationships with zoonotic viral diversity (electronic supplementary material, table S13). Our best model for total number of viruses contained citations, latitude and range area (table 2 and electronic supplementary material, table S24). Citation count, latitude and range area all had positive relationships with total viral diversity (electronic supplementary material, table S25). Finally, the amount of residual variation explained by phylogeny in our bat PGLS models increased (electronic supplementary material, tables S12 and S24) relative to models built using the BE [39] tree.

For rodents, using the more recent F&S [42] phylogeny and pPCA in PGLS regressions did not significantly alter the correlates of viral diversity. Rodent species’ traits identified as important in our models are similar to those in Luis *et al*. [21] (tables 1 and 2). Three models had similar support for total number of viruses in rodent species (table 2 and electronic supplementary material, table S20): citations and torpor, citations and range area, and citations and sympatry. Citation count, range area and sympatry all had positive relationships with total viral diversity (electronic supplementary material, table S21). Torpor usage had a negative relationship with viral diversity, where species that were true hibernators or that did not hibernate, but for which there was some evidence of torpor use, carried fewer viruses than species who did not use any form of torpor (electronic supplementary material, table S21). For number of zoonotic viruses, four models had similar support (electronic supplementary material, table S8): citations and sympatry, citations and torpor use, citations and range area, and citations and IUCN status (table 1 and electronic supplementary material, table S8). Citations, range area and sympatry all had positive relationships with number of zoonotic viruses (electronic supplementary material, table S9), while torpor usage and IUCN status had negative relationships with number of zoonotic viruses. That is, species which used some form of torpor or were listed as Vulnerable, carried fewer viruses (electronic supplementary material, table S9). As in [21], phylogeny did not explain any additional variation in rodent PGLS models (electronic supplementary material, tables S8 and S20).

Analysis of the combined bat/rodent data using [42] did not alter the correlates of viral diversity significantly (tables 1 and 2, electronic supplementary material, tables S16 and S28). Best models for both number of zoonotic viruses and total number of viruses contain the same predictors as in [21] (citations, torpor use, taxon sympatry and order), but the best models no longer contain an interaction between order and taxon sympatry (tables 1 and 2, electronic supplementary material, tables S16 and S28). In both models, rodents and species which used torpor carried fewer viruses. Conversely, sympatry and citation count had positive relationships with viral diversity. Again, phylogeny explained more residual variation than in [21].

### 3.4. An alternative modelling framework

When we adopted an alternative modelling framework using VIFs to remove collinear variables and including all remaining traits in PGLS models to examine relative effect sizes, citation count was often the only significant predictor in models (electronic supplementary material, tables S30–S35). However, in the PGLS model for total number of viruses carried by bats, both citation count and litter size were significant (electronic supplementary material, table S33). Citation count had a positive relationship with total viral diversity, while litter size had a negative relationship (figure 1). For number of zoonotic viruses in bats, citation count, mass and longevity were significant predictors in the PGLS model (electronic supplementary material, table S32). Both citation count and mass scaled positively with zoonotic viral diversity, while longevity scaled negatively (figure 1). As above, phylogeny explained some of the residual variation in the PGLS model for zoonotic viruses in bats (*λ* = 1; electronic supplementary material, table S32), but in contrast to above, did not explain any of the residual variation in the PGLS model for total viral richness (*λ* < 0.0001; electronic supplementary material, table S33).

For rodents, citation count was the only significant predictor in models for number of zoonotic and total number of viruses carried by species (tables S30–S31). In both models, citation count had a positive relationship with rodent viral diversity (figure 1). Additionally, phylogeny did not explain any additional variation in rodent PGLS models (*λ* < 0.0001; electronic supplementary material, tables S30 and S31).

For the combined bat and rodent PGLS model examining total viral richness, citation count was the only significant predictor (electronic supplementary material, table S35) and had a positive relationship with total viral richness (electronic supplementary material, figure S7). For number of zoonotic viruses in the bat/rodent combined data, citations, sympatry and no torpor use were all significant terms (electronic supplementary material, table S34). All of these traits had a positive relationship with number of zoonotic viruses (electronic supplementary material, figure S7). In both PGLS models, phylogeny explained some additional residual variation (*λ* = 0.1762; electronic supplementary material, table S34 and *λ* = 0.2735; electronic supplementary material, table S35).

## 4. Discussion

The importance of species relationships in trait-based studies is well appreciated in the field of comparative biology. In our reanalysis of the data presented in Luis *et al.* [21], we find that for both bats and rodents, when we controlled for phylogeny during preliminary data transformations (i.e. principal component analyses), our first phylogenetic principal component explained the majority of life-history trait variation for bats, rodents and the bat/rodent combined data. Although PC1 was also important in Luis *et al.*, without phylogenetic correction an additional three to four principal components were included and yet together still explained less variation in life-history data for bats, rodents, and bats and rodents combined [21]. Further, as anticipated, but in contrast to [21], we find that phylogeny does have a significant effect in models examining species' trait correlates of viral diversity in bats, both when using the original BE [39] tree and using a more recent, comprehensive phylogeny for bats [41]. For rodents, like [21], we find a minimal impact of phylogeny. Irrespective of differences in the importance of species’ relationships, our best models for viral infection in rodents and bat/rodent combined data, contain similar predictors (i.e. sympatry, citations and torpor use) to those identified by Luis *et al*. Using a more recent phylogeny for bats [41], we find that geographical distribution (i.e. latitude and range area) may be important for explaining patterns of total viral richness, and life-history traits (i.e. lifespan, litter size, litters per year, body mass) may explain patterns of zoonotic viral richness in bats. However, the effects of these ecological predictors are marginal relative to citation count. In conjunction with our assessment of standardized effect sizes, this suggests that ecological traits may explain reservoir status, but that clarifying their influence requires a greater sampling effort of viral diversity across understudied bat and rodent species.

We find a significant phylogenetic signal in all but one of our models for bats, regardless of phylogeny used (electronic supplementary material, tables S10, S12, S22, S24, S32 and S33). We found phylogeny had little effect in only our PGLS model for total viral richness when all ecological predictors were included (electronic supplementary material, table S33). Previous work has found that phylogeny explains little residual variance in the relationship between viral richness and ecological traits in bats [16,20,21]. However, these authors note that this pattern is counterintuitive given the significant links between phylogeny and parasite richness [31]. We demonstrate that phylogeny does indeed have a significant effect in models examining ecological trait correlates of viral diversity in bats, which is in line with comparative studies of other traits in this group (e.g. [33–36]). The differences between our results and those of Luis *et al.* [21], specifically lower *R*^2^ values, different ecological trait correlates and the presence of phylogenetic signal for bats, could be due to the fact that we applied a phylogenetic PCA and may also be influenced by small, unknown differences in the underlying R-scripts.

For rodents, our reanalysis did not alter the importance of phylogeny, bolstering the inference that species relationships are less important for explaining residual variation in rodent reservoir status. Although it is difficult to postulate why phylogeny does not impact the residual error structure in our models for rodents, we can speculate on what may drive lack of signal within patterns of viral richness and ecological traits in rodents (electronic supplementary material, table S2). Given the challenges associated with extensively characterizing viral diversity, to date researchers have probably sampled only a small proportion of the viruses harboured by wild mammals [55]. Considering that the phylogenetic signal in samplings of species may differ from that of the broader group [29], lack of signal could be a function of the subset of rodent species included in this analysis. Conversely, it could be that rodents exhibit greater ecological trait variation through time and as a function of geographical region. Although comparative analyses often rely on species' averages or measures taken from a single population at a single time, not accounting for intraspecific character variation may introduce errors in comparative studies [29,56]. This possibility has been posited to explain low phylogenetic signal in, for example, behavioural characters (e.g. [57]). Given the wide variety of evolutionary process that may influence and generate similar patterns of phylogenetic signal in species’ traits [27,58], future work could look to characterize the processes that may underlie patterns of viral sharing within specific rodent species or geographical regions (e.g. [59–61]).

Our results highlight that the phylogenetic tree used for comparative inference can impact species' trait correlates of viral infection. Although the BE [39] supertree is often used in comparative studies (e.g. [20,21,62]), this tree has numerous polytomies [63], poor resolution closer to the tips (particularly for Muridae, the largest family of rodents) and fails to recover approximately 30% of nodes that are well supported in a mammalian tree constructed using maximum likelihood and Bayesian methods [63]. Conversely, the S&R [41] bat phylogeny is fully resolved (i.e. contains no polytomies) and has been time calibrated using 29 genes (20 376 base pairs for 812 species from one order; compared to 66 genes and 51 089 base pairs used for 4510 species in 26 mammalian orders in [39]), making this phylogeny ideal for addressing comparative questions in bats [41]. We find that, regardless of the tree used, species’ trait correlates of zoonotic and total number of viruses were similar for rodents and in the bat/rodent combined data. However, for bats, the tree used mattered. We found that latitude and range area were both important for predicting total and zoonotic viral diversity using the BE [39] tree, but that using the S&R [41] phylogeny, life-history traits instead predicted number of zoonotic viruses, while geographical traits remained important for predicting total number of viruses. This would suggest that while exposure may be important for the overall number of viruses hosted by a bat species, as has been seen in patterns of pathogen sharing in primates [64] and patterns of viral diversity across multiple mammalian orders [22], life-history traits may be important for governing pathogen evolution that may facilitate host jumping and cross-species emergence.

With a phylogenetic principal components analysis (pPCA), we were able to explain most of the variation (more than 99%) in the life-history traits of bats and rodents with a single phylogenetic principal component (pPC1). Again, this emphasizes the need to appropriately account for species' relationships throughout comparative analyses [27]. Further, using the updated F&S [42] and S&R [41] phylogenies, pPC1 partitioned species of bats and rodents into those with fast (i.e. smaller body size, increased litter size and litters per year, decreased longevity) or slow (i.e. larger bodied, longer lifespans, fewer offspring and litters per year) life histories. Although the applicability of a single, fast–slow continuum across mammal species has been called into question (e.g. [65]), the partitioning of bat species’ along this axis is particularly surprising given that bats, as a whole, are often considered to fall at the slow end of this continuum [66]. However, despite what our pPCA may reveal about the clustering of life-history traits in bats and rodents, pPC1 did not appear in the majority of models examining correlates of total and zoonotic viral richness. Although pPC1 was present in the best model of zoonotic viral richness in bats, its effect size was marginal relative to citation count. This suggests that overall, life-history traits are not important for explaining patterns of viral diversity in bats and rodents. This inference is surprising, given that life-history traits are thought to regulate, to an extent, pathogen dynamics in natural populations. For example, multiple birthing pulses are important for driving filovirus dynamics in bat populations [19,67]. However, when we used VIFs to remove collinear traits and included all remaining predictors in PGLS models (instead of using a PCA) several life-history traits (i.e. litter size, lifespan and mass) exhibited some, albeit small, effect.

Regardless, both our best and full model approaches confirm the potential importance of ecological traits for predicting patterns of viral richness. The majority of our models contained species' sympatry, which prior work has shown correlates positively with patterns of parasite richness [19,21–23,64,68]. Where our models did not contain sympatry, they often contained geographical range area (e.g. our model for total viral richness in bats), which correlates with species’ sympatry (electronic supplementary material, figure S4–S6), and has also been shown to correlate with parasite richness in other groups of mammals [69,70]. As discussed in previous work (e.g. [21,22]), wide-ranging species or those experiencing greater range overlap with heterospecifics, are more likely to interact with other mammalian species and may occupy a greater variety of habitat types, increasing opportunities for pathogen transmission. In addition to geographical distribution, the majority of our models also contained torpor use, which often had a large, but sometimes variable effect in PGLS models (figure 1 and electronic supplementary material, figure S7). Torpor use correlates negatively with viral diversity: species that do not use torpor carry a greater number of viruses. Luis *et al.* [21] also find torpor usage to be important, but note that this pattern is contrary to expectation [21]. Reduced temperatures and metabolic rates associated with torpor correspond to decreased periods of immune functioning, which would be expected to increase infection risk [71,72]. In fact, periodic rewarming during hibernation may be necessary to re-activate immune responses [71]. However, rates of viral replication may also be lowered during torpor use [13]. This, paired with lower contact rates during periods of inactivity [21] may limit the establishment of viral infections in species who use torpor. However, as Luis *et al.* note there is a current lack of understanding about what may drive the relationship between torpor use and viral persistence in reservoir species [21], highlighting the need for additional research.

Although we may be able to make broad inferences about the potential importance of ecological traits for driving patterns of viral richness in reservoir species, our reanalysis emphasizes that patterns of viral diversity are largely driven by citation count. In most of our PGLS regressions, citation count was often the only significant term and had a much larger effect size relative to ecological traits. This was particularly true for bats, where ecological traits exhibited marginal effects relative to citation count (e.g. electronic supplementary material, tables S12, S14, etc.). Further, in our PGLS models containing all ecological predictors, although citation count did not always have the largest standardized effect size, its effect was consistent and not as variable as other traits (i.e. lack of torpor use or IUCN status) (figure 1 and electronic supplementary material, figure S7). Broadly, this suggests that although ecological traits may increase the amount of variation explained by PGLS models, patterns are largely driven by sampling effort, as has been seen in a variety of comparative studies of parasite richness (e.g. [20,21,23,70]). Accurate identification of reservoirs thus, unsurprisingly, requires sampling of understudied species and incorporation of more data into future comparative studies.

While there are barriers to including more data in comparative analyses, such as a lack of natural history information for many species, the importance of species-level citation count brings to light that some of the patterns in this dataset could also be artefacts of the subset of species sampled. Bats and rodents are the two most speciose mammalian orders, comprising well over 60% of living mammalian diversity (approx. 2277 species of rodents [73] and approx. 1300 species of bats [74]). Despite this, the dataset analysed here compared only 66 species of bats (approx. 6% of extant bat diversity) and 81 species of rodents (approx. 4% of extant rodent diversity). As such, caution must be taken when drawing conclusions on a very small sampling of the diversity in Rodentia and Chiroptera. Recently, Han *et al.* [68] used a novel method that overcomes the challenges of missing data common in comparative datasets to predict viral reservoirs across all species of rodents. A study examining order-wide correlates of viral diversity in bats is still lacking, but methods refined and applied by Han *et al*. [19,68] should provide a good starting point.

We examined how methodological choices influence the outcomes of phylogenetic comparative analyses examining species' trait correlates of viral diversity. However, modern phylogenetic methods allow us to do much more than use phylogeny as a statistical control. They allow us to appreciate the evolutionary history of species, better understand patterns of biological diversity, trace character traits back through evolutionary time and make inferences about the evolution of those traits. For example, studies have used phylogeny to examine the risk of host pathogen shifts in primates [64] and predict species’ responses to anthropogenic stressors, such as deforestation [75]. With continued disease emergence from zoonotic reservoirs, modern phylogenetic methods and incorporation of species' relationships into comprehensive trait-based studies could allow for a better understanding of the interplay between ecological and evolutionary drivers of viral diversity. Further, this understanding could aid in the mitigation of future zoonotic outbreaks. As such, trait-based studies offer an important tool for better understanding the ecological drivers of viral diversity in reservoir species, an understanding that can help direct disease surveillance and management efforts.

## Supplementary Material

Supplementary Tables and Figures

## Data accessibility

All data used in our analyses were taken from electronic supplementary material, table S14 of Luis *et al*. [21]. Code for analyses has been deposited in Dryad: http://dx.doi.org/10.5061/dryad.994f813 [76].
