# Supplementary material for: Phylogeny matters: revisiting ‘a comparison of bats and rodents as reservoirs of zoonotic viruses’
Source: R Soc Open Sci. 2019 Feb 13;6(2):181182. doi: 10.1098/rsos.181182 (PMC6408376; doi:10.1098/rsos.181182)
Supplement: Supplementary Tables and Figures [file rsos181182supp1.pdf]

Supplementary Tables & Figures

Phylogenetic Signal and pPCAs

Table S1 Estimates of Pagel's lambda (λ) for bat species' traits based on the Bininda-Emonds *et al.* [1] and Shi & Rabosky phylogenetic trees [2]. Values of λ close to 1 indicate that species' traits are related to shared branch lengths, while values close to 0 indicate traits are phylogenetically independent. Significant values of λ are bolded.

Bat Species' Traits	Bininda-Emonds *et al.*		Shi & Rabosky	
	λ	p-value	λ	p-value
Mass	1.000	< **0.001**	1.000	< **0.001**
Litter Size	1.000	< **0.001**	0.859	< **0.001**
Litters / year	1.000	< **0.001**	0.604	1.0
Longevity	0.000	1.0	0.319	< **0.001**
Migration	0.818	0.032	1.000	**0.003**
Range Area	0.357	0.056	0.512	**0.036**
Sympatry	0.687	< **0.001**	0.686	< **0.001**
Latitude	0.690	< **0.001**	0.838	< **0.001**
Torpor Use	0.669	< **0.001**	1.000	< **0.001**
Citations	0.000	0.992	0.000	1.0
Zoonotic Virus	0.671	< **0.001**	1.000	< **0.001**
Total Virus	0.469	< **0.001**	0.963	< **0.001**

Table S2 Estimates of Pagel's lambda (λ) for rodent species' traits based on the Bininda-Emonds *et al.* [1] and Faurby and Svenning [3] phylogenetic trees. Values of λ close to 1 indicate that species' traits are related to shared branch lengths, while values close to 0 indicate traits are phylogenetically independent. Significant values of λ are bolded.

Rodent Species' Traits	Bininda-Emonds *et al.*		Faurby and Svenning	
	λ	p-value	λ	p-value
Mass	1.000	< **0.001**	1.000	< **0.001**
Litter Size	0.872	< **0.001**	0.784	< **0.001**
Litters / year	0.563	< **0.001**	0.329	< **0.001**
Longevity	0.770	< **0.001**	0.776	< **0.001**
Range Area	0.000	1.000	0.000	1.000
Sympatry	0.201	0.194	0.378	0.084
Latitude	0.950	< **0.001**	0.944	< **0.001**
Torpor Use	1.025	< **0.001**	1.000	< **0.001**
Citations	0.054	0.427	0.060	0.256
Zoonotic Virus	0.017	0.792	0.012	0.792
Total Virus	0.138	0.154	0.067	0.258

Table S3 Estimates of Pagel's lambda (λ) for combined bat/rodent species' traits based on the Bininda-Emonds *et al.* [1] and Faurby and Svenning [3] phylogenetic trees. Values of λ close to 1 indicate species traits are related to shared branch lengths, while values close to 0 indicate traits are phylogenetically independent. Significant values of λ are bolded.

Combined Species' Traits	Bininda-Emonds *et al.*		Faurby and Svenning	
	λ	p-value	λ	p-value
Mass	1.000	**< 0.001**	1.00	**< 0.001**
Litter Size	0.946	**< 0.001**	0.899	**< 0.001**
Litters / year	1.000	**< 0.001**	0.492	**< 0.001**
Longevity	0.450	**< 0.001**	0.628	**< 0.001**
Range Area	0.000	1.000	0.000	1.000
Sympatry	0.442	**< 0.001**	0.559	**< 0.001**
Latitude	0.942	**< 0.001**	0.927	**< 0.001**
Torpor Use	0.873	**< 0.001**	0.971	**< 0.001**
Citations	0.111	**0.020**	0.098	**0.006**
Zoonotic Virus	0.467	**0.000**	0.369	**< 0.001**
Total Virus	0.347	**0.002**	0.295	**0.003**

Table S4 Loading values of the first principal component (pPC1) from a phylogenetic principal components analysis using the Bininda-Emonds *et al.* [1] phylogenetic trees for bats and rodents.

Species' Traits	pPC_1		
	Rodents	Bats	Combined
Mass	-0.311	-0.205	-0.205
Longevity	-1.000	-1.000	-1.000
Litters / year	0.063	0.172	0.069
Litter Size	-0.037	0.108	0.004
Proportion of Variance Explained	0.999999	0.999999	0.999999

Table S5 Loading values of the first principal component (pPC1) from a phylogenetic principal components analysis using the Shi and Rabosky [2] and the Faurby and Svenning [3] phylogenetic trees.

Species' Traits	pPC_1		
	Rodents	Bats	Combined
Mass	-0.143	-0.173	-0.125
Longevity	-1.000	-1.000	-1.000
Litters / year	0.164	0.056	0.008
Litter Size	0.184	-0.299	-0.055
Proportion of Variance Explained	0.999999	0.999999	0.999999

Species Trait Correlates of Zoonotic Viral Diversity

Table S6 Rankings of rodent PGLS models with number of zoonotic viruses as the response. Estimates for λ were calculated using the Bininda-Emonds *et al.* [1] phylogenetic tree.

Models	AIC_c	ΔAIC_c	R^2	λ	p-value
zvirus ~ citations + sympatry + IUCN	314.4	0	0.36	0.000	<0.0001
zvirus ~ citations + sympatry	314.6	0.2	0.35	0.000	<0.0001
zvirus ~ citations + sympatry + latitude	315.9	1.5	0.35	0.000	<0.0001
zvirus ~ citations + sympatry + torpor	315.9	1.5	0.36	0.000	<0.0001
zvirus ~ citations + IUCN	319.9	5.5	0.30	0.000	<0.0001
zvirus ~ citations + torpor	320.9	6.5	0.31	0.000	<0.0001
zvirus ~ citations + IUCN + pPC1	321.7	7.4	0.29	0.000	<0.0001
zvirus ~ citations	321.8	9.1	0.27	0.000	<0.0001
zvirus ~ citations + area	323.5	11.1	0.27	0.000	<0.0001
zvirus ~ citations + area + latitude	325.5	19.6	0.26	0.000	<0.0001
zvirus ~ sympatry	334	29.8	0.14	0.000	0.0006
The null (intercept model)	344.2	0	NA	0.017	NA

Table S7 Details of the best rodent PGLS model for number of zoonotic viruses, built using the Bininda-Emonds *et al.* [1] phylogenetic tree (zvirus ~ citations + sympatry).

	Coefficients	Standard Error	p-value
(Intercept)	-2.56	0.83	0.0028
Citations	0.82	0.17	<0.0001
Sympatry	0.005	0.002	0.003

Table S8 Rankings of rodent PGLS models with number of zoonotic viruses as the response. Estimates for λ were calculated using the Faurby and Svenning [3] phylogenetic tree.

Models	AIC_c	ΔAIC_c	R^2	λ	p-value
zvirus ~ citations + area	359.6	0	0.30	0.000	<0.0001
zvirus ~ citations + sympatry	360.4	0.8	0.30	0.000	<0.0001
zvirus ~ citations + IUCN	360.7	1.1	0.29	0.000	<0.0001
zvirus ~ citations + sympatry + IUCN	360.9	1.3	0.30	0.000	<0.0001
zvirus ~ citations + sympatry + torpor	360.9	1.3	0.31	0.000	<0.0001
zvirus ~ citations + torpor	361.0	1.4	0.30	0.000	<0.0001
zvirus ~ citations + area + latitude	361.6	2	0.30	0.000	<0.0001
zvirus ~ citations + sympatry + latitude	362.3	2.7	0.29	0.000	<0.0001
zvirus ~ citations	362.3	2.7	0.27	0.000	<0.0001
zvirus ~ citations + IUCN + pPC1	362.7	3.1	0.29	0.000	<0.0001
zvirus ~ sympatry	385.8	26.2	0.03	0.008	0.0777
The null (intercept model)	387.0	27.4	NA	0.012	NA

Table S9 Details of the best rodent PGLS models for number of zoonotic viruses, built using the Faurby and Svenning [3] phylogenetic tree (zvirus ~ citations + torpor, zvirus ~ citations + area, zvirus ~ citations + sympatry, and zvirus ~ citations + IUCN).

	Coefficients	Standard Error	p-value
(Intercept)	-1.81	0.80	0.027
Citations	0.93	0.16	<0.0001
Torpor: Some Use	-0.82	0.74	0.266
Torpor: Hibernation	-1.38	0.64	0.034

	Coefficients	Standard Error	p-value
(Intercept)	-6.24	2.09	0.004
Citations	0.82	0.16	<0.0001
Area	0.30	0.14	0.033

	Coefficients	Standard Error	p-value
(Intercept)	-5.43	1.91	0.006
Citations	0.89	0.16	<0.0001
Sympatry	0.7	1.96	0.053

	Coefficients	Standard Error	p-value
(Intercept)	-2.13	0.80	0.009
Citations	0.94	0.16	<0.0001
IUCN	-2.92	1.55	0.06

Table S10 Rankings of bat PGLS models with number of zoonotic viruses as the response. Estimates for λ were calculated using the Bininda-Emonds *et al.* [1] phylogenetic tree.

Models	AIC_c	ΔAIC_c	R^2	λ	p-value
zvirus ~ citations + latitude + area	243.9	0	0.14	0.806	0.0029
zvirus ~ citations + sympatry + latitude	247.7	3.8	0.24	0.619	0.0006
zvirus ~ citations + sympatry	248.2	4.3	0.22	0.675	0.0005
zvirus ~ citations + sympatry + pPC1	250.2	6.3	0.20	0.675	0.0019
zvirus ~ citations + sympatry + IUCN	251.0	7.1	0.20	0.665	0.0032
zvirus ~ citations + sympatry + torpor	251.2	7.3	0.20	0.605	0.0033
zvirus ~ sympatry	252.9	9	0.13	0.615	0.0029
zvirus ~ citations	253.0	9.1	0.13	0.699	0.0033
zvirus ~ citations + pPC1	254.6	10.7	0.12	0.694	0.0112
zvirus ~ citations + pPC1 + migration	258.5	14.6	0.09	0.695	0.062
The null (intercept model)	260.1	16.2	NA	0.671	NA

Table S11 Details of the best bat PGLS model for number of zoonotic viruses, built using the Bininda-Emonds *et al.* [1] phylogenetic tree (zvirus ~ citations + latitude + area).

	Coefficients	Standard Error	p-value
(Intercept)	0.67	1.45	0.644
Citations	0.59	0.26	0.028
Latitude	-0.04	0.018	0.022
Area	0.0001	0.00003	0.005

Table S12 Rankings of bat PGLS models with number of zoonotic viruses as the response. Estimates for λ were calculated using the Shi and Rabosky [2] phylogenetic tree.

Models	AIC_c	ΔAIC_c	R^2	λ	p-value
zvirus ~ citations + pPC1	248.3	0	0.16	1.000	0.0022
The null (intercept model)	254.3	6	NA	1.000	NA
zvirus ~ citations + latitude +area	269.4	21.1	0.24	0.986	0.0002
zvirus ~ citations + sympatry	272.0	23.7	0.20	0.951	0.0004
zvirus ~ citations + sympatry + pPC1	272.9	24.6	0.19	0.986	0.0013
zvirus ~ citations + sympatry + latitude	273.0	24.7	0.20	0.943	0.0010
zvirus ~ citations	273.9	25.6	0.13	1.000	0.0019
zvirus ~ citations + sympatry + torpor	274.7	26.4	0.20	0.928	0.0022
zvirus ~ citations + sympatry + IUCN	275.8	27.5	0.18	0.949	0.0040
zvirus ~ citations + pPC1 + migration	276.9	28.6	0.15	1.000	0.010
zvirus ~ sympatry	280.7	32.4	0.07	0.957	0.0227

Table S13 Details of the best bat PGLS model for number of zoonotic viruses, built using the Shi and Rabosky [2] phylogenetic tree (zvirus ~ citations + pPC1).

	Coefficients	Standard Error	p-value
(Intercept)	-1.21	1.58	0.448
Citations	1.08	0.30	0.0007
pPC1	0.0002	0.0001	0.102

Table S14 Rankings of combined bat and rodent PGLS models with number of zoonotic viruses as the response. Estimates for λ were calculated using the Bininda-Emonds *et al.* [1] phylogenetic tree.

Models	AIC_c	ΔAIC_c	R^2	λ	p-value
zvirus ~ citations + sympatry	562	0	0.32	0.607	<0.0001
zvirus ~ order + citations + sympatry + torpor	563.5	1.5	0.32	0.457	<0.0001
zvirus ~ order + citations + sympatry	563.0	1	0.32	0.564	<0.0001
zvirus ~ order*torpor + citations + sympatry	567.4	5.4	0.31	0.44	<0.0001
zvirus ~ order*sympatry + citations + area	564.1	2.1	0.32	0.528	<0.0001
zvirus ~ order*sympatry + citations + torpor + latitude	564.1	2.1	0.41	0.303	<0.0001
zvirus ~ order*sympatry + citations	564.4	2.4	0.31	0.521	<0.0001
zvirus ~ order + citations + sympatry + pPC1	564.8	2.8	0.31	0.564	<0.0001
zvirus ~ order*sympatry + citations + torpor	565.4	3.4	0.41	0.351	<0.0001
zvirus ~ order*sympatry + order*citations + torpor	565.7	3.7	0.32	0.367	<0.0001
zvirus ~ order*sympatry + citations + latitude	566.3	4.3	0.31	0.535	<0.0001
zvirus ~ order + citations + sympatry + IUCN	566.4	4.4	0.31	0.566	<0.0001
zvirus ~ citations + order*sympatry + order*torpor	567.9	5.9	0.40	0.271	<0.0001
zvirus ~ order + citations	577.4	15.4	0.23	0.539	<0.0001
zvirus ~ order + citations + latitude*torpor	580.8	18.8	0.24	0.391	<0.0001
zvirus ~ order + sympatry	590	28	0.15	0.423	<0.0001
The null (intercept model)	608.9	46.9	NA	0.467	NA

Table S15 Details of the best combined bat and rodent PGLS model for number of zoonotic viruses, built using the Bininda-Emonds *et al.* [1] phylogenetic tree (zvirus ~ citations + sympatry).

	Coefficients	Standard Error	p-value
(Intercept)	-2.33	1.02	0.024
Citations	0.84	0.15	<0.0001
Sympatry	0.007	0.002	<0.0001

Table S16 Rankings of combined bat and rodent PGLS models with number of zoonotic viruses as the response. Estimates for λ were calculated using the Faurby and Svenning [3] phylogenetic tree.

Models	AIC_c	ΔAIC_c	R^2	λ	p-value
zvirus ~ order + citations + sympatry + torpor	640.8	0	0.29	0.286	<0.0001
zvirus ~ order*sympatry + citations + torpor	643.6	2.8	0.36	0.252	<0.0001
zvirus ~ order + citations + sympatry	643.4	2.6	0.27	0.376	<0.0001
zvirus ~ citations + sympatry	643.4	2.6	0.27	0.438	<0.0001
zvirus ~ order*sympatry + citations + torpor + latitude	645.4	4.6	0.36	0.261	<0.0001
zvirus ~ order* sympatry + order*citations + torpor	645.5	4.7	0.35	0.252	<0.0001
zvirus ~ order*torpor + citations + sympatry	645.5	4.7	0.36	0.250	<0.0001
zvirus ~ order*sympatry + citations	645.4	4.6	0.26	0.372	<0.0001
zvirus ~ order + citations + sympatry + pPC1	645.4	4.6	0.26	0.375	<0.0001
zvirus ~ order*sympatry + citations + area	646.4	5.6	0.26	0.379	<0.0001
zvirus ~ order + citations + sympatry + IUCN	647.1	6.3	0.26	0.383	<0.0001
zvirus ~ order*sympatry + citations + latitude	647.3	6.5	0.26	0.361	<0.0001
zvirus ~ citations + order*sympatry + order*torpor	647.3	6.5	0.36	0.214	<0.0001
zvirus ~ order + citations	648.5	7.7	0.24	0.447	<0.0001
zvirus ~ order + citations + latitude*torpor	650.8	10	0.25	0.325	0.0581
zvirus ~ order + sympatry	678.4	37.6	0.07	0.249	0.0026
The null (intercept model)	685.5	44.7	NA	0.369	NA

Table S17 Details of the best combined bat and rodent PGLS model for number of zoonotic viruses, built using the Faurby and Svenning [3] phylogenetic tree (zvirus ~ order + citations + sympatry + torpor).

	Coefficients	Standard Error	p-value
(Intercept)	-2.96	1.67	0.080
Order Rodentia	-2.40	1.20	0.048
Citations	0.98	0.144	<0.0001
Sympatry	0.65	0.266	0.016
Torpor: Some Use	-1.00	0.583	0.088
Torpor: Hibernation	-1.38	0.614	0.026

Species Trait Correlates of Total Viral Diversity

Table S18 Rankings of rodent PGLS models with total number of viruses as the response. Estimates for λ were calculated using the Bininda-Emonds *et al.* [1] phylogenetic tree.

Models	AIC_c	ΔAIC_c	R^2	λ	p-value
tvirus ~ citations + sympatry + torpor	381.4	0	0.47	0.000	<0.0001
tvirus ~ citations + sympatry	382.3	0.9	0.45	0.000	<0.0001
tvirus ~ citations + sympatry + latitude	384.2	2.8	0.44	0.000	<0.0001
tvirus ~ citations + sympatry + IUCN	384.3	2.9	0.44	0.000	<0.0001
tvirus ~ citations + torpor	385.9	4.5	0.43	0.000	<0.0001
tvirus ~ citations	388.9	7.5	0.37	0.093	<0.0001
tvirus ~ citations + IUCN	390.7	9.3	0.38	0.060	<0.0001
tvirus ~ citations + area	390.9	9.5	0.36	0.118	<0.0001
tvirus ~ citations + IUCN + pPC1	392.6	11.2	0.37	0.113	<0.0001
tvirus ~ citations + area + latitude	392.9	11.5	0.37	0.142	<0.0001
tvirus ~ sympatry	413.6	32.2	0.12	0.086	0.002
The null (intercept model)	421.7	40.3	NA	0.138	NA

Table S19 Details of the best rodent PGLS model for total number of viruses, built using the Bininda-Emonds *et al.* [1] phylogenetic tree (tvirus ~ citations + sympatry).

	Coefficients	Standard Error	p-value
(Intercept)	-5.79	1.31	<0.0001
Citations	1.72	0.27	<0.0001
Sympatry	0.008	0.003	0.004

Table S20 Rankings of rodent PGLS models with total number of viruses as the response. Estimates for λ were calculated using the Faurby and Svenning [3] phylogenetic tree.

**Models**	AIC_c	ΔAIC_c	R^2	λ	p-value
tvirus ~ citations + torpor	433.5	0	0.40	0.000	<0.0001
tvirus ~ citations + sympatry + torpor	433.9	0.4	0.40	0.000	<0.0001
tvirus ~ citations + area	435.0	1.5	0.38	0.000	<0.0001
tvirus ~ citations + sympatry	435.1	1.6	0.38	0.000	<0.0001
tvirus ~ citations	436.4	2.9	0.36	0.000	<0.0001
tvirus ~ citations + area + latitude	436.9	3.4	0.38	0.000	<0.0001
tvirus ~ citations + sympatry + IUCN	437.1	3.6	0.37	0.000	<0.0001
tvirus ~ citations + sympatry + latitude	437.1	3.6	0.37	0.000	<0.0001
tvirus ~ citations + IUCN	438.1	4.6	0.36	0.000	<0.0001
tvirus ~ citations + IUCN + pPC1	440.1	6.6	0.35	0.000	<0.0001
tvirus ~ sympatry	470.4	36.9	0.02	0.069	0.1270
The null (intercept model)	470.8	37.3	NA	0.067	NA

Table S21 Details of the best rodent PGLS models for total number of viruses, built using the Faurby and Svenning [3] phylogenetic tree (tvirus ~ citations + torpor, tvirus ~ citations + area, tvirus ~ citations + sympatry).

	Coefficients	Standard Error	p-value
(Intercept)	-4.25	1.25	0.0011
Citations	1.80	0.25	<0.0001
Torpor: Some Torpor	-1.95	1.15	0.0933
Torpor: Hibernation	-2.24	1.00	0.0283

	Coefficients	Standard Error	p-value
(Intercept)	-10.25	3.33	0.0029
Citations	1.65	0.26	<0.0001
Area	0.40	0.22	0.073

	Coefficients	Standard Error	p-value
(Intercept)	-9.54	3.02	0.0023
Citations	1.74	0.25	<0.0001
Sympatry	1.01	0.57	0.079

Table S22 Rankings of bat PGLS models with total number of viruses as the response. Estimates for λ were calculated using the Bininda-Emonds *et al.* [1] phylogenetic tree.

Models	AIC_c	ΔAIC_c	R^2	λ	p-value
tvirus ~ citations +latitude + area	286.1	0	0.33	0.688	<0.0001
tvirus ~ citations + sympatry	287.8	1.7	0.28	0.522	<0.0001
tvirus ~ citations + sympatry + pPC1	289.1	3	0.28	0.517	0.0002
tvirus ~ citations + sympatry + torpor	289.1	3	0.29	0.477	0.0002
tvirus ~ citations	289.5	3.4	0.25	0.580	<0.0001
tvirus ~ citations + sympatry + latitude	289.7	3.6	0.27	0.496	0.0002
tvirus ~ citations + sympatry + IUCN	291.1	5	0.26	0.515	0.0005
tvirus ~ citations + pPC1	291.5	5.4	0.24	0.581	0.0003
tvirus ~ citations + pPC1 + migration	295.2	9.1	0.21	0.571	0.0025
tvirus ~ sympatry	299.5	13.4	0.11	0.319	0.007
The null (intercept model)	304.5	13.4	NA	0.469	NA

Table S23 Details of the best bat PGLS model for total number of viruses, built using the Bininda-Emonds *et al.* [1] phylogenetic tree phylogenetic tree (tvirus ~ citations + sympatry).

	Coefficients	Standard Error	p-value
(Intercept)	-3.24	1.97	0.106
Citations	1.51	0.38	0.0002
Sympatry	0.009	0.005	0.06

Table S24 Rankings of bat PGLS models with total number of viruses as the response. Estimates for λ were calculated using the Shi and Rabosky [2] phylogenetic tree.

Models	AIC_c	ΔAIC_c	R^2	λ	p-value
tvirus ~ citations + latitude + area	288.5	0	0.32	1.000	<0.0001
tvirus ~ citations + pPC1	292.1	3.6	0.26	1.000	<0.0001
tvirus ~ citations + sympatry + latitude	292.1	3.6	0.28	1.000	<0.0001
tvirus ~ citations + sympatry + pPC1	293.1	4.6	0.28	1.000	<0.0001
tvirus ~ citations + sympatry + IUCN	294.1	5.6	0.27	1.000	0.0002
tvirus ~ citations + sympatry	312.4	23.9	0.29	1.000	<0.0001
tvirus ~ citations	318.4	29.9	0.26	1.000	<0.0001
tvirus ~ citations + sympatry + torpor	318.4	29.9	0.30	1.000	<0.0001
tvirus ~ citations + pPC1 + migration	323.3	34.8	0.24	1.000	0.0004
tvirus ~ sympatry	335.3	46.8	0.05	0.842	0.0497
The null (intercept model)	336.9	48.4	NA	0.962	NA

Table S25 Details of the best bat PGLS model for total number of viruses, built using the Shi and Rabosky [2] phylogenetic tree phylogenetic tree (tvirus ~ citations + latitude + area).

	Coefficients	Standard Error	p-value
(Intercept)	-14.81	5.21	0.006
Citations	1.72	0.24	0.0000
Latitude	0.009	0.02	0.613
Area	0.75	0.33	0.026

Table S26 Rankings of combined bat and rodent PGLS models with total number of viruses as the response. Estimates for λ were calculated using the Bininda-Emonds *et al.* [1] phylogenetic tree.

Models	AIC_c	ΔAIC_c	R^2	λ	p-value
tvirus ~ order*sympatry + citations + torpor	664.9	0	0.45	0.000	<0.0001
tvirus ~ order + citations + sympatry + torpor	665.0	0.1	0.45	0.000	<0.0001
tvirus ~ order*sympatry + citations + torpor + latitude	665.9	1	0.45	0.000	<0.0001
tvirus ~ order* sympatry + order* citations + torpor	666.6	1.7	0.44	0.000	<0.0001
tvirus ~ citations + sympatry	667.9	3	0.38	0.398	<0.0001
tvirus ~ order + citations + sympatry	668.2	3.3	0.38	0.316	<0.0001
tvirus ~ order*torpor + citations + sympatry	668.5	3.6	0.44	0.000	<0.0001
tvirus ~ citations + order*sympatry + order*torpor	668.8	3.9	0.44	0.000	<0.0001
tvirus ~ order + citations + sympatry + pPC1	669.5	4.6	0.38	0.331	<0.0001
tvirus ~ order*sympatry + citations	670	5.1	0.38	0.291	<0.0001
tvirus ~ order*sympatry + citations + area	671.0	6.1	0.38	0.317	<0.0001
tvirus ~ order + citations + sympatry + IUCN	671.5	6.6	0.37	0.314	<0.0001
tvirus ~ order*sympatry + citations + latitude	671.8	6.9	0.37	0.331	<0.0001
tvirus ~ order + citations	678	13.1	0.33	0.420	<0.0001
tvirus ~ order + citations + latitude*torpor	678.2	13.3	0.40	0.114	<0.0001
tvirus ~ order + sympatry	712.5	47.6	0.13	0.214	<0.0001
The null (intercept model)	727.4	62.5	NA	0.347	NA

Table S27 Details of the best combined bat and rodent PGLS model for total number of viruses, built using the Bininda-Emonds *et al.* [1] phylogenetic tree phylogenetic tree (tvirus ~ order + citations + sympatry + torpor).

	Coefficients	Standard Error	p-value
(Intercept)	-2.86	1.00	0.005
Order: Rodentia	-2.51	0.59	<0.0001
Citations	1.73	0.22	<0.0001
Sympatry	0.009	0.002	0.0002
Torpor: Some Torpor	-1.82	0.765	0.0186
Torpor: Hibernation	-1.92	0.680	0.0055

Table S28 Rankings of combined bat and rodent PGLS models with total number of viruses as the response. Estimates for λ were calculated using the Faurby and Svenning [3] phylogenetic tree.

Models	AIC_c	ΔAIC_c	R^2	λ	p-value
tvirus ~ order + citations + sympatry + torpor	756.7	0	0.40	0.209	<0.0001
tvirus ~ order*sympatry + citations + torpor	758.5	1.8	0.40	0.175	<0.0001
tvirus ~order + citations + sympatry	759.3	2.6	0.35	0.327	<0.0001
tvirus ~ citations + sympatry	759.6	2.9	0.34	0.405	<0.0001
tvirus ~ order*torpor + citations + sympatry	760.6	3.9	0.40	0.200	<0.0001
tvirus ~ order*sympatry + citations + torpor + latitude	760.3	3.6	0.40	0.196	<0.0001
tvirus ~ order*sympatry + order* citations + torpor	760.5	3.8	0.40	0.178	<0.0001
tvirus ~ order + citations + sympatry + pPC1	761.1	4.4	0.34	0.330	<0.0001
tvirus ~ order*sympatry + citations	761.3	4.6	0.34	0.329	<0.0001
tvirus ~ citations + order*sympatry + order*torpor	762.4	5.7	0.40	0.168	<0.0001
tvirus ~ order + citations	762.6	6.1	0.33	0.409	<0.0001
tvirus ~ order*sympatry + citations + area	762.7	6	0.34	0.323	<0.0001
tvirus ~ order + citations + sympatry + IUCN	763.1	6.4	0.34	0.320	<0.0001
tvirus ~ order*sympatry + citations + latitude	763.3	6.6	0.34	0.328	<0.0001
tvirus ~order + citations + latitude*torpor	764.4	7.7	0.38	0.234	<0.0001
tvirus ~ order + sympatry	812.2	55.5	0.06	0.179	<0.0001
The null (intercept model)	817.6	60.9	NA	0.295	NA

Table S29 Details of the best combined bat and rodent PGLS model for total number of viruses, built using the Faurby and Svenning [3] phylogenetic tree (tvirus ~ order + citations + sympatry + torpor).

	Coefficients	Standard Error	p-value
(Intercept)	-6.42	2.14	0.003
Order: Rodentia	-2.97	0.61	<0.0001
Citations	1.80	0.21	<0.0001
Sympatry	1.05	0.40	0.009
Torpor: Some Torpor	-1.95	0.81	0.02
Torpor: Hibernation	-2.19	0.71	0.002

Correlates of Viral Diversity for an Alternate Modeling Framework

Table S30 Details of a full PGLS model for the number of zoonotic viruses carried by rodents, built using the Faurby and Svenning [3] phylogenetic tree (log zvirus ~ sympatry + area + citations + mass + latitude + torpor + longevity + litters/year + litter size + IUCN). All ecological predictors were standardized prior to model fitting. Significant predictors are marked by an (*). The model had an adjusted $R^2 = 0.28$ and $p < 0.0001$. All p values are conditional on $\lambda < 0.0001$.

	Coefficients	Standard Error	p-value
(Intercept)	0.73	0.20	0.0006*
Range Area	0.07	0.10	0.457
Citations	0.20	0.09	0.026*
Litters/year	0.03	0.09	0.753
Litter Size	0.12	0.10	0.225
Mass	-0.13	0.09	0.171
Sympatry	0.02	0.09	0.801
Latitude	0.06	0.08	0.440
IUCN: Vulnerable	-0.45	0.46	0.329
Torpor: No Torpor	0.34	0.23	0.153
Torpor: Torpor	0.10	0.30	0.737
Longevity	0.19	0.10	0.065

Table S31 Details of a full PGLS model for the total number of viruses carried by rodents, built using the Faurby and Svenning [3] phylogenetic tree (log tvirus ~ sympatry + area + citations + mass + latitude + torpor + longevity + litters/year + litter size + IUCN). All ecological predictors were standardized prior to model fitting. Significant predictors are marked by an (*). The model had an adjusted $R^2 = 0.32$ and p <0.0001. All p values are conditional on λ <0.0001.

	Coefficients	Standard Error	p-value
(Intercept)	0.56	0.25	0.026*
Range Area	0.14	0.12	0.231
Citations	0.37	0.10	0.001*
Litters/year	0.18	0.11	0.109
Litter Size	0.16	0.12	0.193
Mass	-0.05	0.11	0.653
Sympatry	0.01	0.11	0.906
Latitude	0.11	0.09	0.227
IUCN: Vulnerable	0.61	0.55	0.273
Torpor: No Torpor	0.38	0.28	0.184
Torpor: Torpor	0.18	0.36	0.621
Longevity	0.15	0.12	0.242

Table S32 Details of a full PGLS model for the number of zoonotic viruses carried by bats, built using the Shi and Rabosky [2] phylogenetic tree (log zvirus ~ sympatry + area + citations + mass + longevity + litters/year + litter size + IUCN + migration). All ecological predictors were standardized prior to model fitting. Significant predictors are marked by an (*). The model had an adjusted $R^2 = 0.28$ and $p < 0.0001$. All p values are conditional on $\lambda = 1$.

	Coefficients	Standard Error	p-value
(Intercept)	1.09	0.26	0.0001*
Range Area	0.11	0.07	0.151
Citations	0.31	0.08	0.0004*
Litters/year	0.09	0.08	0.257
Litter Size	-0.11	0.08	0.179
Mass	0.35	0.13	0.009*
Sympatry	0.02	0.08	0.815
IUCN: Near Threatened	-0.15	0.19	0.427
IUCN: Vulnerable	0.30	0.50	0.555
Migration: No Migration	0.13	0.22	0.565
Migration: Regional Migrant	0.24	0.23	0.306
Longevity	-0.15	0.07	0.038*

Table S33 Details of a full PGLS model for the total number of viruses carried by bats, built using the Shi and Rabosky [2] phylogenetic tree (log tvirus ~ sympatry + area + citations + mass + longevity + litters/year + litter size + IUCN + migration). All ecological predictors were standardized prior to model fitting. Significant predictors are marked by an (*). The model had an adjusted $R^2 = 0.48$ and $p < 0.0001$. All p values are conditional on $\lambda < 0.0001$.

	Coefficients	Standard Error	p-value
(Intercept)	1.12	0.29	0.0003*
Range Area	0.12	0.13	0.358
Citations	0.49	0.13	0.0005*
Litters/year	0.09	0.11	0.441
Litter Size	-0.29	0.11	0.012*
Mass	0.17	0.13	0.177
Sympatry	0.18	0.13	0.176
IUCN: Near Threatened	-0.23	0.33	0.492
IUCN: Vulnerable	0.09	0.88	0.915
Migration: No Migration	-0.05	0.32	0.875
Migration: Regional Migrant	0.14	0.35	0.699
Longevity	-0.05	0.13	0.698

Table S34 Details of a full PGLS model for the number of zoonotic viruses carried by bats and rodents, built using the Faurby and Svenning [3] phylogenetic tree (log zvirus ~ sympatry + area + citations + mass + latitude + longevity + litters/year + litter size + IUCN + torpor). All ecological predictors were standardized prior to model fitting. Significant predictors are marked by an (*). The model had an adjusted $R^2 = 0.32$ and $p < 0.0001$. All p values are conditional on $\lambda = 0.1762$.

	Coefficients	Standard Error	p-value
(Intercept)	0.89	0.16	<0.0001*
Range Area	0.06	0.06	0.334
Citations	0.29	0.06	<0.0001*
Litters/year	-0.004	0.08	0.949
Litter Size	-0.10	0.10	0.289
Mass	-0.10	0.07	0.168
Sympatry	0.12	0.06	0.032*
Latitude	0.01	0.06	0.962
IUCN: Near Threatened	-0.04	0.24	0.860
IUCN: Vulnerable	0.05	0.29	0.873
Torpor: No Torpor	0.35	0.17	0.036*
Torpor: Torpor	0.13	0.17	0.435
Longevity	0.05	0.07	0.504

Table S35 Details of a full PGLS model for the total number of viruses carried by bats and rodents, built using the Faurby and Svenning [3] phylogenetic tree (log tvirus ~ sympatry + area + citations + mass + latitude + longevity + litters/year + litter size + IUCN + torpor). All ecological predictors were standardized prior to model fitting. Significant predictors are marked by an (*). The model had an adjusted $R^2 = 0.42$ and $p < 0.0001$. All p values are conditional on $\lambda = 0.2735$.

	Coefficients	Standard Error	p-value
(Intercept)	0.85	0.23	0.0004*
Range Area	0.12	0.08	0.123
Citations	0.50	0.08	<0.0001*
Litters/year	0.14	0.10	0.144
Litter Size	-0.22	0.13	0.109
Mass	-0.06	0.10	0.551
Sympatry	0.13	0.07	0.076
Latitude	0.06	0.08	0.421
IUCN: Near Threatened	-0.19	0.31	0.547
IUCN: Vulnerable	0.68	0.38	0.075
Torpor: No Torpor	0.19	0.22	0.393
Torpor: Torpor	-0.10	0.22	0.662
Longevity	0.06	0.09	0.539

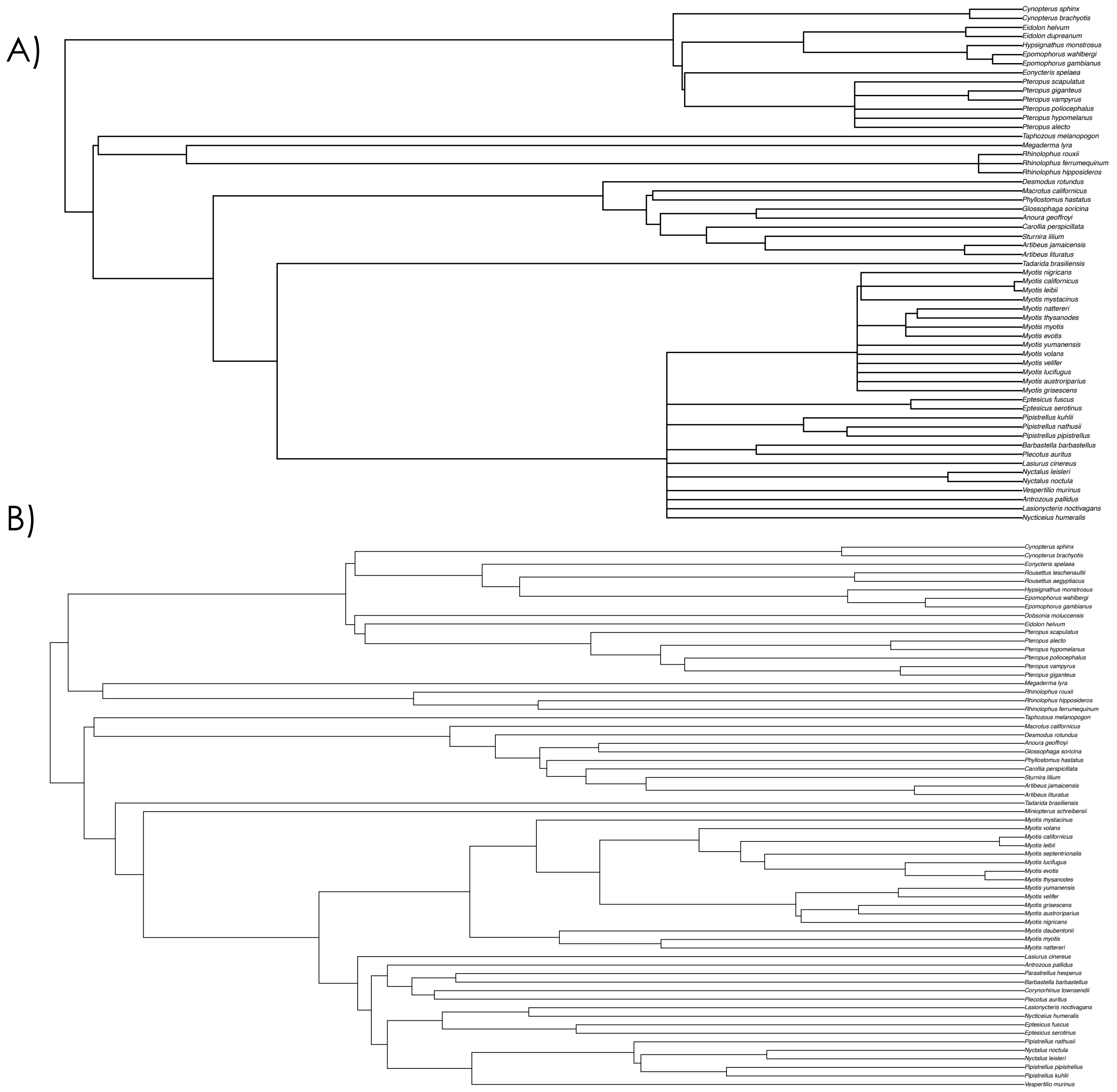

Figure S1: Phylogenies used for PGLS analyses of zoonotic and total viral diversity in bats. A) Bininda-Emonds *et al.* phylogenetic tree [1] and B) Shi & Rabosky phylogenetic tree [2].

C)

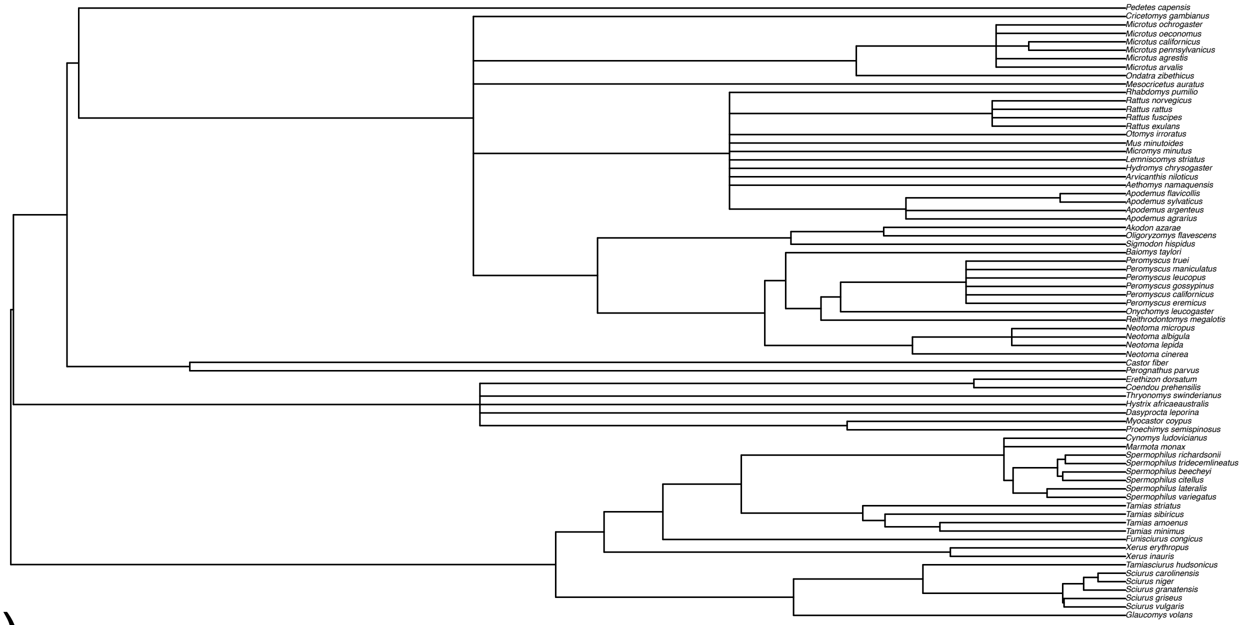

D)

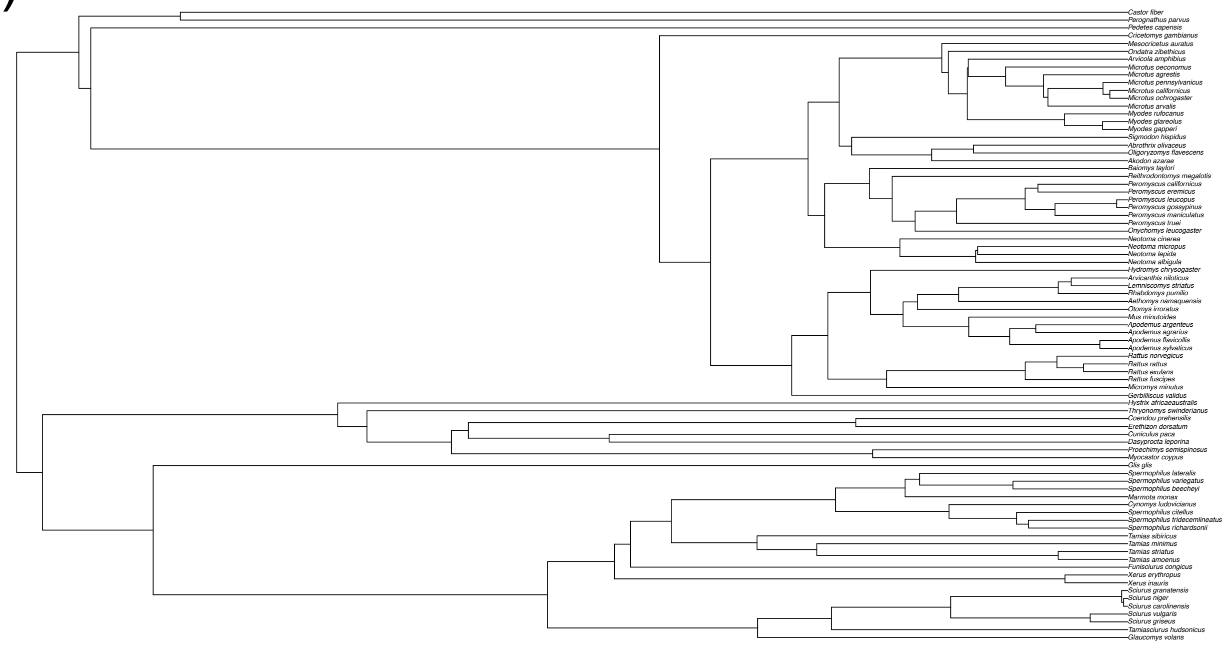

Figure S2: Phylogenies used for PGLS analyses of zoonotic and total viral diversity in rodents. C) Bininda-Emonds *et al.* phylogenetic tree [1] and D) Faurby & Svenning phylogenetic tree [3].

E)

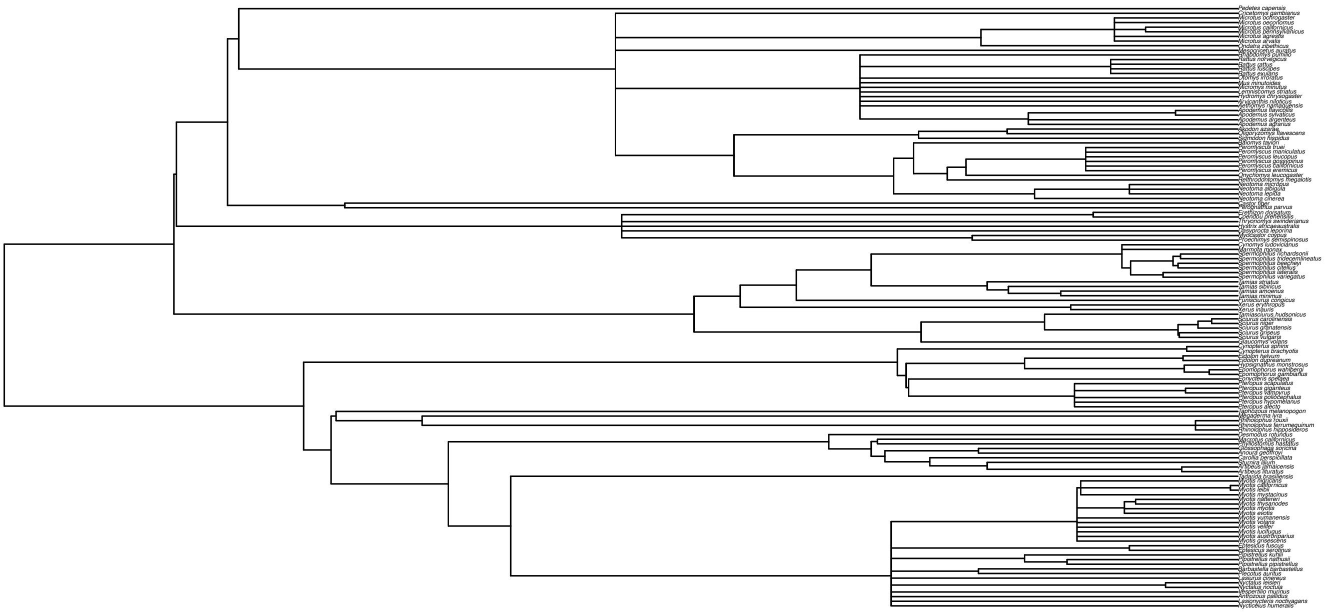

F)

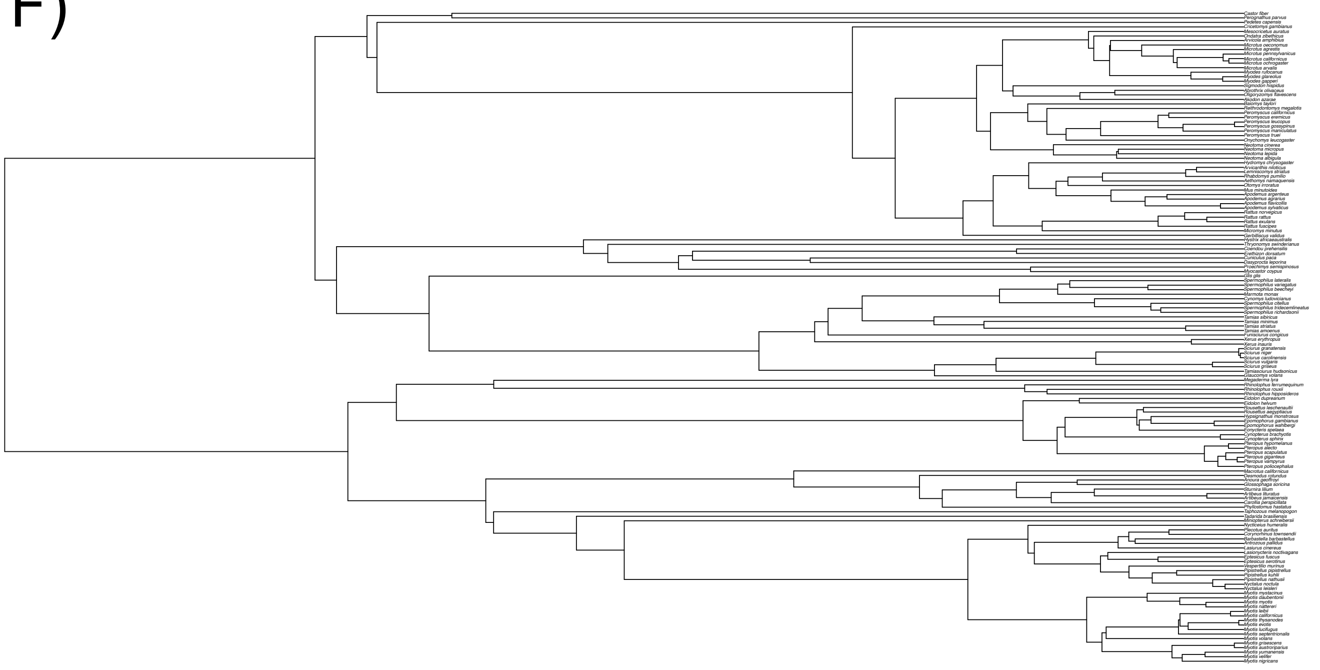

Figure S3: Phylogenies used for PGLS analyses of zoonotic and total viral diversity in the combined bat and rodent data. E) Bininda-Emonds *et al.* phylogenetic tree [1] and F) Faurby & Svenning phylogenetic tree [3]

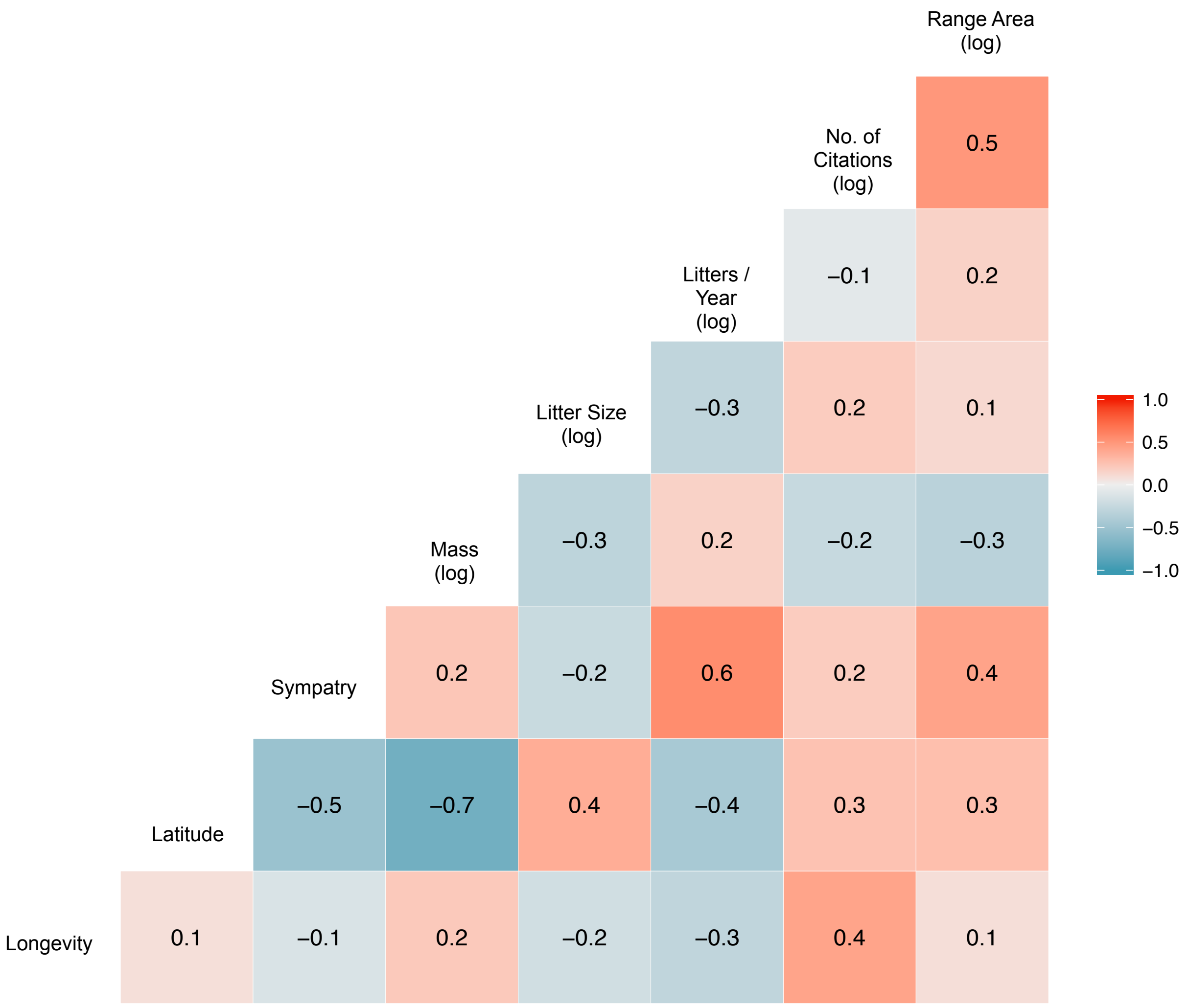

Figure S4: Correlation matrix for continuous ecological traits included in PGLS analyses of zoonotic and total viral diversity in bats.

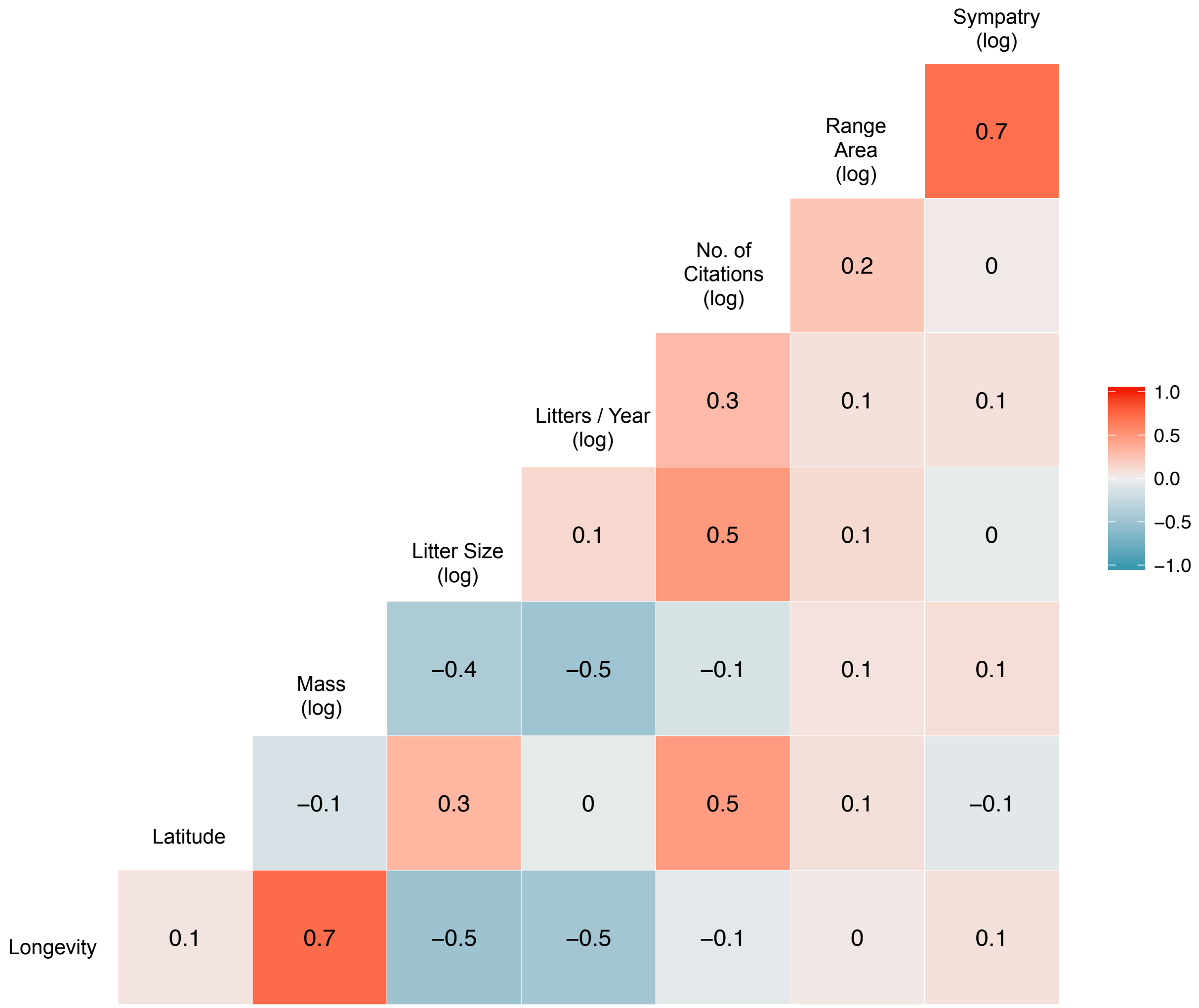

Figure S5: Correlation matrix for continuous ecological traits included in PGLS analyses of zoonotic and total viral diversity in rodents.

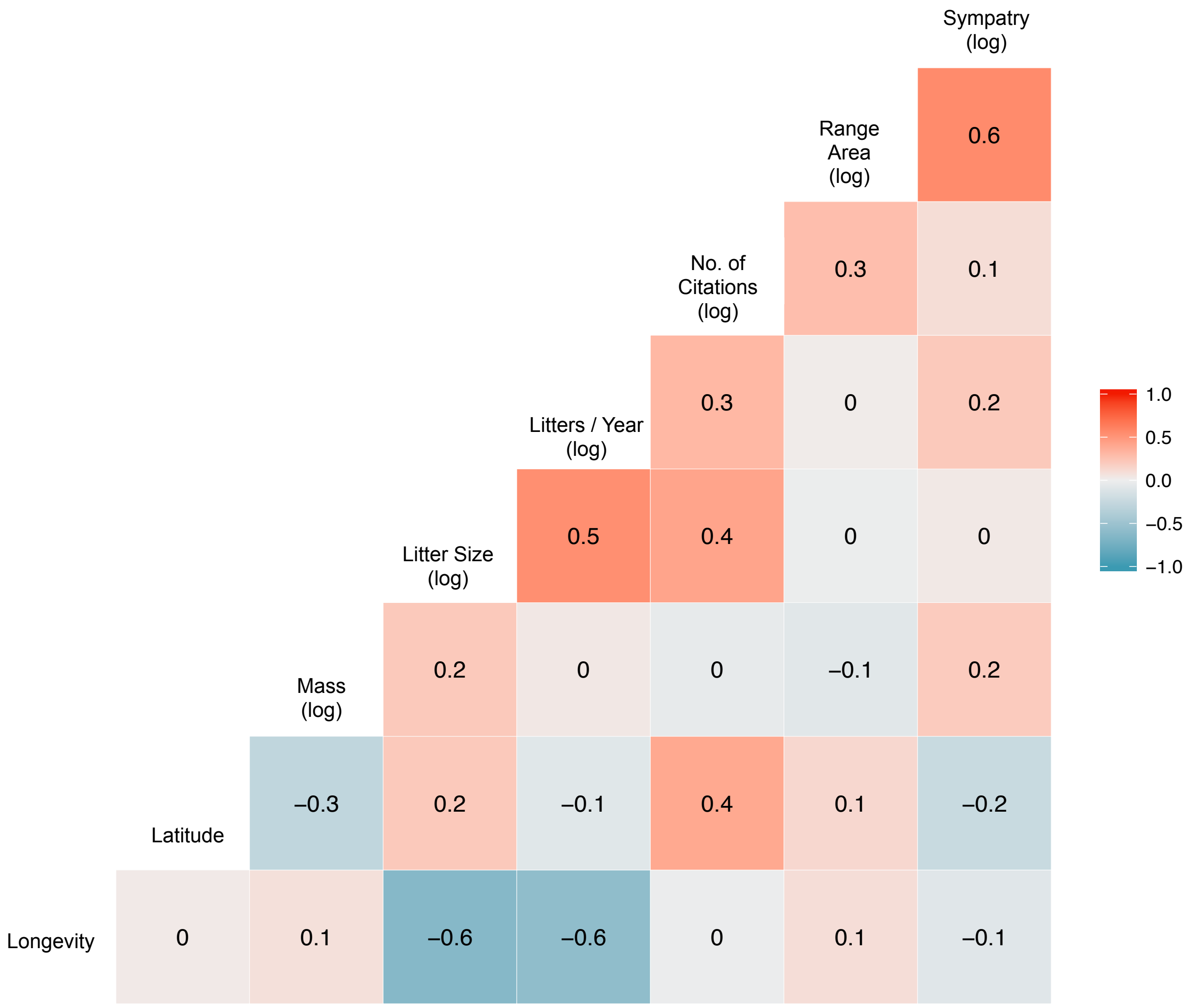

Figure S6: Correlation matrix for continuous ecological traits included in PGLS analyses of zoonotic and total viral diversity in the bat/rodent combined data.

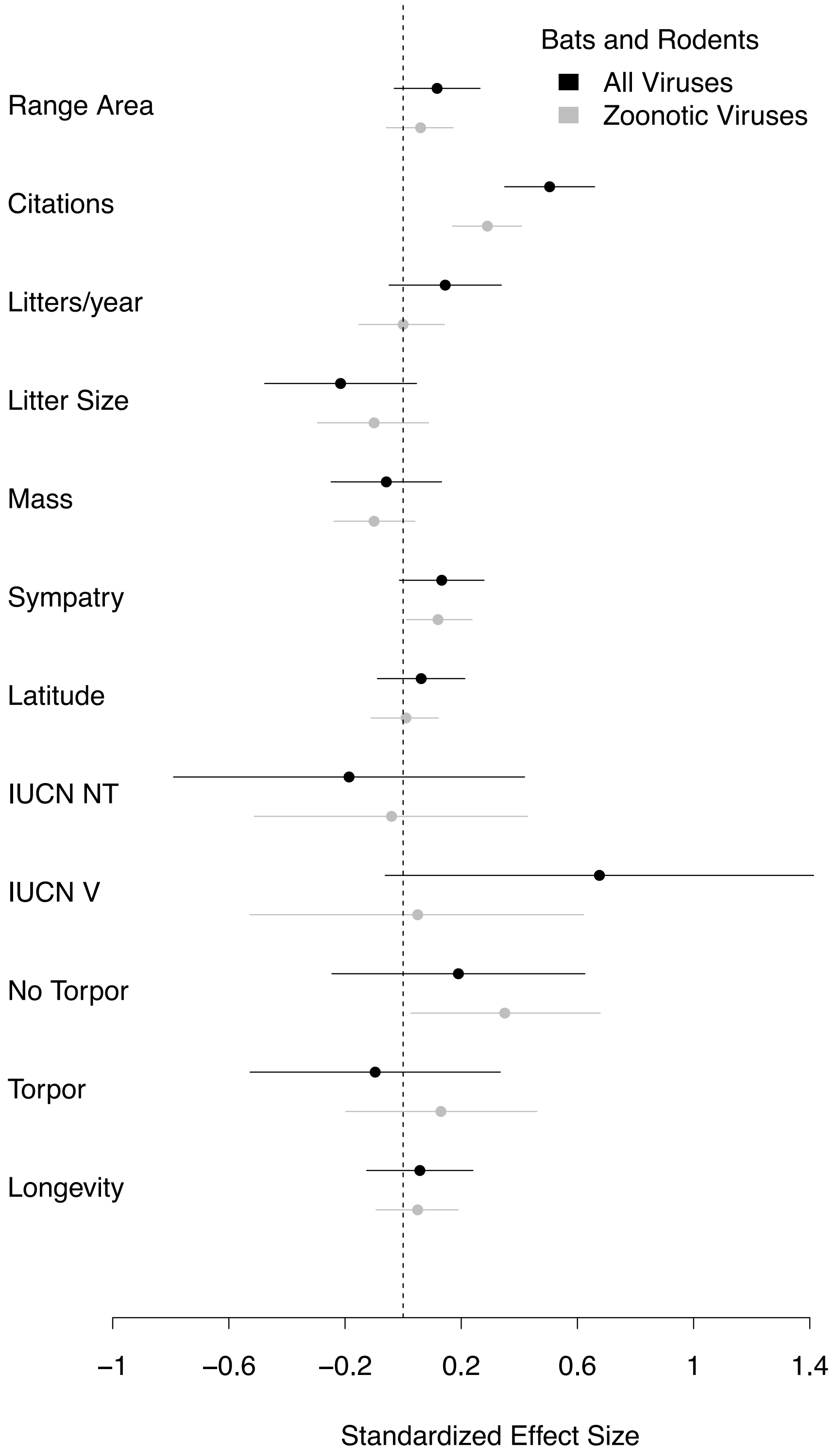

Figure S7: Plot of standardized effect size from PGLS models including all ecological traits with variance inflation factors ≤ 5 (instead of pPC1). Black dots represent the standardized effect size of traits for models examining correlates of total viral richness in the bat/rodent combined data. Gray dots represent standardized effect size of traits for PGLS models of zoonotic viral diversity in the bat/rodent combined data. Error bars represent 95% confidence intervals. The dashed line indicates an effect size of zero.

References:

1. Bininda-Emonds ORP *et al.* 2007 The delayed rise of present-day mammals. *Nature* **446**, 507–512. (doi:10.1038/nature05634)

2. Shi JJ, Rabosky DL. 2015 Speciation dynamics during the global radiation of extant bats. *Evolution* **69**, 1528–1545. (doi:10.1111/evo.12681)

3. Faurby S, Svenning JC. 2015 A species-level phylogeny of all extant and late Quaternary extinct mammals using a novel heuristic-hierarchical Bayesian approach. *Mol. Phylogenet. Evol.* **84**, 14–26. (doi:10.1016/j.ympev.2014.11.001)
